# Bile does not increase the aggressiveness of pancreatic fluid towards fibrin-based sealants in a short-term *ex-vivo* setting

**DOI:** 10.1007/s00423-026-04300-2

**Published:** 2026-10-06

**Authors:** Nora Katharina Drees, Julian Malzkorn, Britta Majchrzak-Stiller, Johanna Strotmann, David Wiedemann, Chris Braumann, Waldemar Uhl, Philipp Höhn

**Affiliations:** 1 Medical Faculty, Ruhr - University Bochum, Bochum, Germany; 2 Department of General and Visceral Surgery, St. Josef - Hospital, Ruhr - University Bochum, Bochum, Germany; 3 Department of General, Visceral and Vascular Surgery, Evangelische Kliniken Gelsenkirchen, Akademisches Lehrkrankenhaus der Universität Duisburg-Essen, Gelsenkirchen, Germany

**Keywords:** Pancreas, Bile, POPF, POBF, Fibrin sealant

## Abstract

**Purpose:**

Postoperative pancreatic fistulas are among the most serious complications of pancreatic surgery. Biliary fistulas, though usually less severe, can occur after pancreaticoduodenectomy and, when combined with pancreatic fistulas, increase morbidity and mortality. This study is the first to investigate, whether bile enhances the harmful effects of pancreatic fluid on sealant integrity in a short-term *ex vivo* model using a fibrin-based sealant, thereby contributing to fistula formation.

**Methods:**

We developed and validated an improved ex-vivo setup to simulate the conditions during pancreatic surgery: the physiological incubation bio-simulator 2 (PIBS 2). A defined defect in porcine pleura was sealed with the fibrin sealant TachoSil^®^ and exposed to various fluids from human origin (pancreatic fluid, bile fluid and a combination of both) for 60 min in a physiological environment. Electrolyte solution served as control. We evaluated the overall leakage rates and time to leakage. Results were plotted as Kaplan-Meier survival curves and analyzed using the log-rank test (Mantel-Cox) and Fisher’s exact test.

**Results:**

PIBS 2 achieved stable defect closure for the electrolyte solution. Pancreatic fluid demonstrated the highest leakage rates (75%) and significantly impaired sealant stability (*p* < 0.001), whereas bile fluid alone showed no significant effect on sealant integrity (*p* = 0.63). When combined, bile and pancreatic fluid resulted in a significantly lower leakage rate compared to pancreatic fluid alone (47% vs. 75%, *p* = 0.041; odds ratio 3.31).

**Conclusion:**

This study replicates previous results, showing that pancreatic fluid is highly aggressive towards fibrin-based sealants. In contrast to clinical findings, the addition of bile did not significantly increase this effect in this ex-vivo model, suggesting that morbidity in patients with combined fistulas may not be primarily caused by increased aggressiveness of pancreatic fluid after its combination with bile.

**Supplementary information:**

The online version contains supplementary material available at https://doi.org/10.1007/s00423-026-04300-2.

## Introduction

Pancreatic surgery is among the most complex and technically demanding procedures in general and visceral surgery [1]. Common indications for pancreaticoduodenectomy (PD) include chronic inflammation as well as malignant or cystic tumors [2]. Despite continuous advances in surgical techniques and perioperative care, postoperative complications following PD substantially increase treatment costs and healthcare resource utilization [3]. Among these, the postoperative pancreatic fistula (POPF) represents a major morbidity and healthcare expenditure with reported postoperative morbidity rates of up to 30–50% and mortality rates of up to 10% [4]. Therefore, effective preventive strategies are essential to reduce financial burden and to improve patient outcome.

In 2016, the International Study Group of Pancreatic Surgery (ISGPS) updated the definition and grading system of POPF. POPF is defined as a drain fluid amylase level exceeding three times the serum amylase activity, accompanied by clinically relevance [5]. An isolated biochemical elevation without clinical impact is classified as a biochemical leak (BL), whereas grade B POPF requires treatment, such as endoscopic or percutaneous drainage. The most severe grade C is characterized by organ failure, an indication for reoperation, or death of the patient due to the fistula [5]. Similarly, postoperative biliary fistula (POBF) is defined as a measured bilirubin level in the drain fluid three times above the plasma bilirubin level and is further classified according to the severity of the leak [6]. Although less frequent, biliary fistula may lead to severe complications including sepsis, multi-organ failure, hemorrhage, and death [5, 7, 8].

The main pathogenic mechanism underlying POPF is enzymatic digestion of intra-abdominal tissue, leading to infected necrosis and bleeding [1, 9]. In biliary fistulas, bacterial infection and digestive effects of biliary acids have been proposed as pathogenic mechanisms. Biliary fluid has also been suggested to modulate this process by promoting the activation of pancreatic enzymes, such as trypsin, an effect that may be further influenced by lipopolysaccharides present in contaminated bile [7]. These observations suggested that interactions between pancreatic fluid (PF) and bile fluid (BF) could amplify tissue-destructive processes. Beyond enzymatic interactions, increasing attention has been directed towards inflammatory pathways, particularly the association between biliary tract infections and the development of POPF after PD [4]. Accordingly, surgical techniques aiming to separate the biliary and pancreatic ducts during PD have been proposed to mitigate the severity of POPF [10, 11].

Since the late 1970s, surgical sealants have been used in hepatobiliary and pancreatic surgery to reinforce pancreatic anastomoses and reduce leakage after PD [12]. Among these, TachoSil^®^ (Takeda, Tokio, Japan) is widely applied. TachoSil^®^ is a collagen patch coated with human fibrinogen and thrombin. Upon contact with blood or liquids, the coagulation factors are released and trigger the final steps of the coagulations cascade, forming a fibrin network that provides local haemostasis and tissue sealing [13]. Due to its standardized patch-based application and its ability to form a mechanically stable network, TachoSil^®^ is used broadly in abdominal surgery to achieve local haemostasis, as well as to seal resection surfaces, anastomoses and vascular sutures. Although TachoSil^®^ is primarily classified as a haemostatic agent, it has been extensively investigated for POPF treatment. However, clinical and experimental studies have not demonstrated a consistent reduction in POPF rates [12, 14]. One proposed explanation is proteolytic degradation of the fibrin matrix by pancreatic enzymes [12, 14, 15].

While POBF is generally associated with limited clinical consequences and is considered less harmful [8], the coexistence of a POPF and POBF is associated with an increase in the overall impact, leading to significantly increased morbidity and mortality [7, 8, 16]. These observations underscore the need to better understand potential interactions between PF and BF.

The primary aim of this study is to investigate whether BF enhances PF–induced degradation of a fibrin-based surgical sealant as a surrogate for overall enzymatic aggressiveness. Building on a previously published and validated experimental model, we established a short-term ex-vivo setup designed to simulate key intraoperative conditions and assess the adhesive performance of biomaterials. The effect of BF on the aggressiveness of PF was evaluated by measuring the mechanical stability of the fibrin-based surgical sealant TachoSil^®^ under standardized and controlled experimental conditions. We hypothesized that PF exposure reduces the mechanical sealant stability of TachoSil^®^, and that concomitant exposure to BF may further impair sealant integrity. By quantifying adhesive strength under defined experimental conditions, we sought to clarify whether BF exerts an additive or synergistic effect on PF-mediated sealant degradation.

## Methods

Based on the previously published physiological incubation bio-simulator (PIBS) developed by our research group [17], the experimental setup of this study was technically refined to improve precision, standardization, and economical use of biological samples. The optimized setup was named PIBS 2.

### Acquisition of pancreatic fluid and bile

We collected pancreatic fluid and bile from intraoperatively placed drains in patients with clinically confirmed postoperative pancreatic or biliary fistula. Informed consent was obtained from all patients prior to fluid collection. Enzymatic activity of amylase, lipase and carboxypeptidase of the PF was determined using commercially available enzyme activity assays performed according to the manufacturers’ instructions (Amylase Activity Assay Kit MAK 009–1KT, Sigma-Aldrich^®^, St. Louis, USA; Lipase Activity Assay Kit II MAK 047–1KT, Sigma-Aldrich^®^, St. Louis, USA; Carboxypeptidase A Assay Kit CS 1130, Sigma-Aldrich^®^, St. Louis, USA). The results are listed in Suppl. Table 1.

### Experimental setup – PIBS 2

Four 50 mL reaction tubes (CELLSTAR^®^ TUBES, Greiner Bio-One International, Austria) were prepared by removing the bottoms and converting the screw caps into fixation rings using a core drill. A thoracic diaphragm of porcine origin served as the biological membrane for analyzing the adhesive strength of TachoSil^®^. The pleural layer was carefully dissected from the diaphragm muscle, cut to size (4 × 4 cm), and stored at 6 °C until preparation within three days at most. Porcine pleura was selected due to its structural stability, minimal autolysis, reproducibility, and ethical availability. Its histological and biomechanical, particularly its elasticity, are similar to those of human pleura, providing a standardized and mechanically consistent biological substrate [18].

**Fig. 1:**
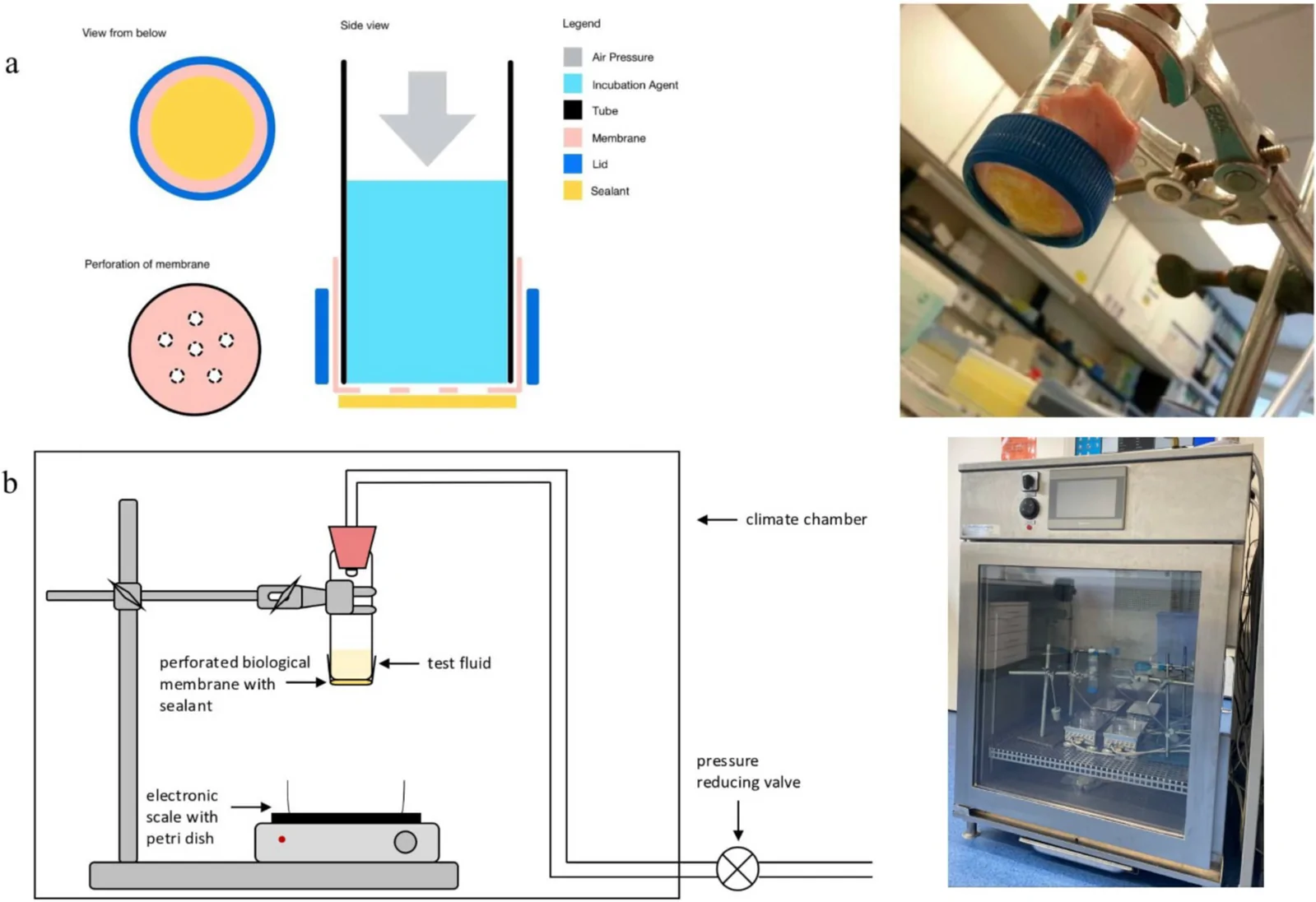
**a**) Experimental setup of the test tubes for testing sealant integrity in the PIBS 2. The image on the left shows a schematic diagram of the experimental setup. The test tubes are covered with perforated porcine pleura, coated with the sealant TachoSil^®^ and filled with a test medium. The view from below demonstrates the perforation of the pleura in more detail, followed by a view of the perorated pleura with the sealant applied. The actual test setup is shown on the right-hand side. **b**) Experimental setup for testing sealant integrity in the PIBS 2 inside the climate chamber. The test fluids are placed above electronic scales with petri dishes within a climate chamber. A user menu allows the precise adjustment and control of the environmental conditions

The membrane was placed over the opening of the test tube and fixed in place by screwing on the plastic ring. Integrity was verified by filling the tubes with 40 ml of electrolyte solution (ES) for 30 s to exclude pre-existing leaks. The membrane was perforated to allow fluid and air permeability. Using a predefined template placed centrally on the membrane, six perforations were made with a 14-G needle (Vasofix^®^ Safety, B Braun, Melsungen, Germany) in the center of the pleura with a diameter of 1 cm (Fig. 1a). The needle was inserted manually at a perpendicular angle of 90°. All punctures were performed by the same investigator using consistent manual pressure. The perforations were arranged symmetrically within a defined circular area to ensure standardized defect geometry. The defect was closed using the fibrin sealant TachoSil^®^ according to the manufacturer’s instructions. The patch was cut to size using a circular template to a diameter of 3 cm. The sealant was positioned centrally over the perforated area, ensuring a uniform circumferential overlap of 1 cm beyond the outermost perforation margin. A compress moistened with ES was applied with gentle manual pressure for 5 min to ensure uniform adhesion. Seal integrity was confirmed by refilling the test tube with 40 mL of ES for 30 s and verifying absence of leakage.

Four test tubes were tested in parallel, filled with 4 ml of test fluid, and secured in a laboratory stand within an incubator (Modell MKKL 200, Labortechnik Vertrieb LinTex, Eutin, Germany). Electronic scales (PCE-SD 0.3 10 × 15 C, PCE Instruments, Meschede, Germany) with Petri dishes on top were placed under each test tube inside the incubator. Separate scales under each test tube enabled precise measurements of the weight of the leaking fluid over time. The top openings of the test tubes were fitted with perforated stoppers (Rotilabo^®^ stopper with bore, natural rubber, Ø bore 6 mm, Ø bottom 25 mm, Oehmen Labortechnik, Bochum, Germany) and connected to rubber tubes (TOL-TX 08/13, Guttasyn, Witten, Germany). These were led out of the incubator and connected to the laboratory’s compressed air wall. An intermediate pressure reducer (pressure reducer type 106; ELGAS, Otterfing, Germany) allowed the application of defined air pressure. The measurement displays were attached outside the incubator (Fig. 1b).

### Test procedure

To evaluate the new experimental setting, tests were performed using 4 mL ES on the intact pleura with and without the sealant TachoSil^®^, followed by ES on a perforated pleura with TachoSil^®^. After successful validation experiments, the TachoSil^®^-sealed tubes were filled with 4 ml of various fluids: pancreatic fluid, bile fluid or electrolyte solution. Furthermore, a combination of the liquids was examined by filling the tubes with 4 ml PF and 4 ml BF. The samples of PF and BF are drainage fluids of human origin. We used 10 different PF samples and 10 different BF samples. Each fluid sample was examined in a multiple determination of three or more measurements. In addition, a positive control was prepared using Kreon 35.000 (420 mg pancreatin per 10 ml). The resulting solution contained 3.500 Ph. Eur. units/ml lipolytic activity, 2.520 Ph. Eur. units/ml amylolytic activity, and 1.400 Ph. Eur. units/ml proteolytic activity. The incubator was set to a constant temperature of 37 °C and humidity of 100%. Similar to intrapancreatic pressure conditions, a pressure of 20 mbar was applied to the liquids via the pressurization system to simulate pancreatic duct pressure [19].

### Test evaluation

Each experiment lasted 60 minutes (min). Leakage was assessed every 2.5 min by recording signs of leakage (stable or unstable defect closure), time to leakage (TTL) and the leakage weight (in grams). Leakage occurring within the first 5 min was defined as dropout [17]. This threshold was chosen as the contact between the sealant and fluid was deemed too short for relevant proteolytic effects to occur and therefore early leakage before positive control was considered most likely attributable to technical failure.

### Microbiological analysis

As part of the routine clinical work-up, intraoperative swabs of the common bile duct (DHC) were obtained and subjected to standard microbiological analysis. The microbiological results were used to characterize bacterial colonization of the biliary tract and were considered as a potential confounding factor in the interpretation of the *ex vivo* experiments. Whenever a sufficient amount of pancreatic fluid was available, a microbiological swab was obtained.

### Statistical analysis

Statistical analysis and data visualisations were performed using R (version 4.5.1) and RStudio (version 2025.09.2), including the packages tidyverse, gt, knitr, survival, ggsurvfit, and rstatix. The occurrence and time to leakage were analysed using Kaplan-Meier curves to estimate probability of stable sealing over time (in min) for the different body fluids. Group comparisons were conducted using the log-rank test (Mantel-Cox). Leakage proportions were compared with Fisher’s exact test. For pairwise log-rank comparisons, p-values were adjusted using the Bonferroni method, and corrected p-values are reported where applicable.

## Results

### Validation of the PIBS 2

**Fig. 2:**
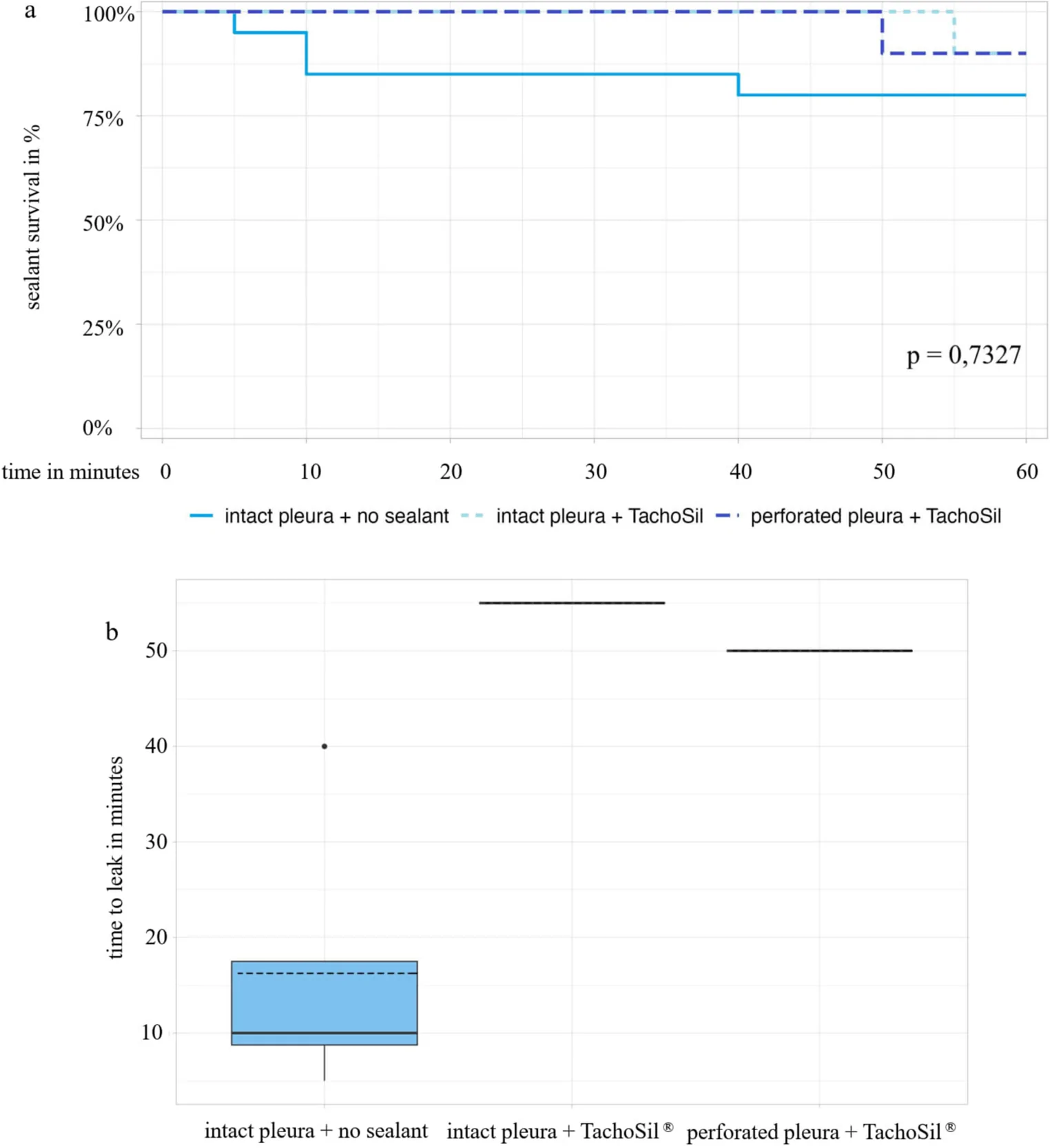
**a**) Kaplan-Meier curves of different pleural conditions in the PIBS 2. The graph illustrates the proportion of intact sealants according to three different settings (intact pleura without a sealant, intact pleural with TachoSil^®^ and perforated pleura with TachoSil^®^) after incubation with ES (ES = electrolyte solution) in the PIBS 2 for 60 minutes. **b**) Boxplot of the TTL for different pleural conditions in the PIBS 2. The boxplots display the TTL (time to leakage) in minutes under three conditions: intact pleura without a sealant, intact pleura treated with TachoSil^®^, and perforated pleura treated with TachoSil^®^. The box represents the interquartile range, the horizontal line indicates the median and the dashed line shows the mean

The PIBS 2 setup was validated under three experimental conditions: intact pleura without a sealant, intact pleura with TachoSil^®^, and perforated pleura with TachoSil^®^ (Table 1). Applying the fibrin sealant TachoSil^®^ on intact and perforated pleurae in the presence of ES resulted in stable defect closure in 90% of cases. When incubating an intact pleura without a sealant in PIBS 2, leakage occurred in four measurements (20%). Overall, no statistically significant difference between the setups were detected (Fisher’s exact test, *p* = 0.73, Fig. 2).

**Table 1:** Leakages of different pleural conditions in the PIBS 2. The table shows the results of three different settings (intact pleura without a sealant, intact pleural with TachoSil^®^ and perforated pleura with TachoSil^®^) after incubation with electrolyte solution in the PIBS 2 for 60 min. Values represent the number of tests performed, the number and percentage of leakages and the average TTL (time to leak) in minutes

setup	tests per protocol	leakage	Ø TTL ± SD
intact pleura + no sealant	20	4 (20%)	16,3 ± 16,01
intact pleura + TachoSil	10	1 (10%)	55
perforated pleura + TachoSil	10	1 (10%)	50
in total	40	6 (15%)	40,4

### Degradation-related effects of various body fluids on a fibrin-based sealant

The effects of four different fluids on the integrity of the fibrin sealant TachoSil^®^ were assessed in 121 measurements. 17 experimental setups showed a measurable leakage within the first 5 min of the experimental procedure and were therefore classified as dropouts, resulting in 104 measurements included in the statistical evaluation. Consequently, the Kaplan–Meier curves do not display any events within the initial 5 min interval. Each fluid sample was tested repeatedly, ensuring that at least one complete evaluable triplicate measurement per sample was included in the final analysis, even if individual tests resulted in dropouts. Table 2 gives an overview of the results.

**Table 2:** Leakage of different body fluids in the PIBS2 The table shows the results of a defect closure using the fibrin sealant TachoSil^®^ when being exposed to four different fluid types in the PIBS2 for 60 min. Values represent the number of tests performed (n), the number and percentage of leakages and the average TTL (time to leakage (in minutes). PF = pancreatic fluid, BF = bile fluid, ES = electrolyte solution (control group). Tests with leakage occurring within 5 min were characterized as dropouts and excluded from the statistical analysis

body fluids	*n*	leakage	Ø TTL (min) ± SD	dropouts
ES	20	2 (10%)	52.5 ± 3.54	0
PF	52	39 (75%)	26.32 ± 12.76	4 (7.14%)
BF	15	3 (20%)	38.33 ± 21.26	7 (31.82%)
PF + BF	17	8 (47.06)	26.69 ± 14.08	6 (26.07%)

ES served as a control condition and showed a leakage rate of 10% (2/20 measurements) and an average TTL of 52.50 min. A positive control with Kreon demonstrated a mean TTL of 20.75 min with a standard deviation of 12.11 min. A preliminary analysis of the pancreatic fluid samples ensured that only active fluids were used (Suppl. Table 1).

**Fig. 3:**
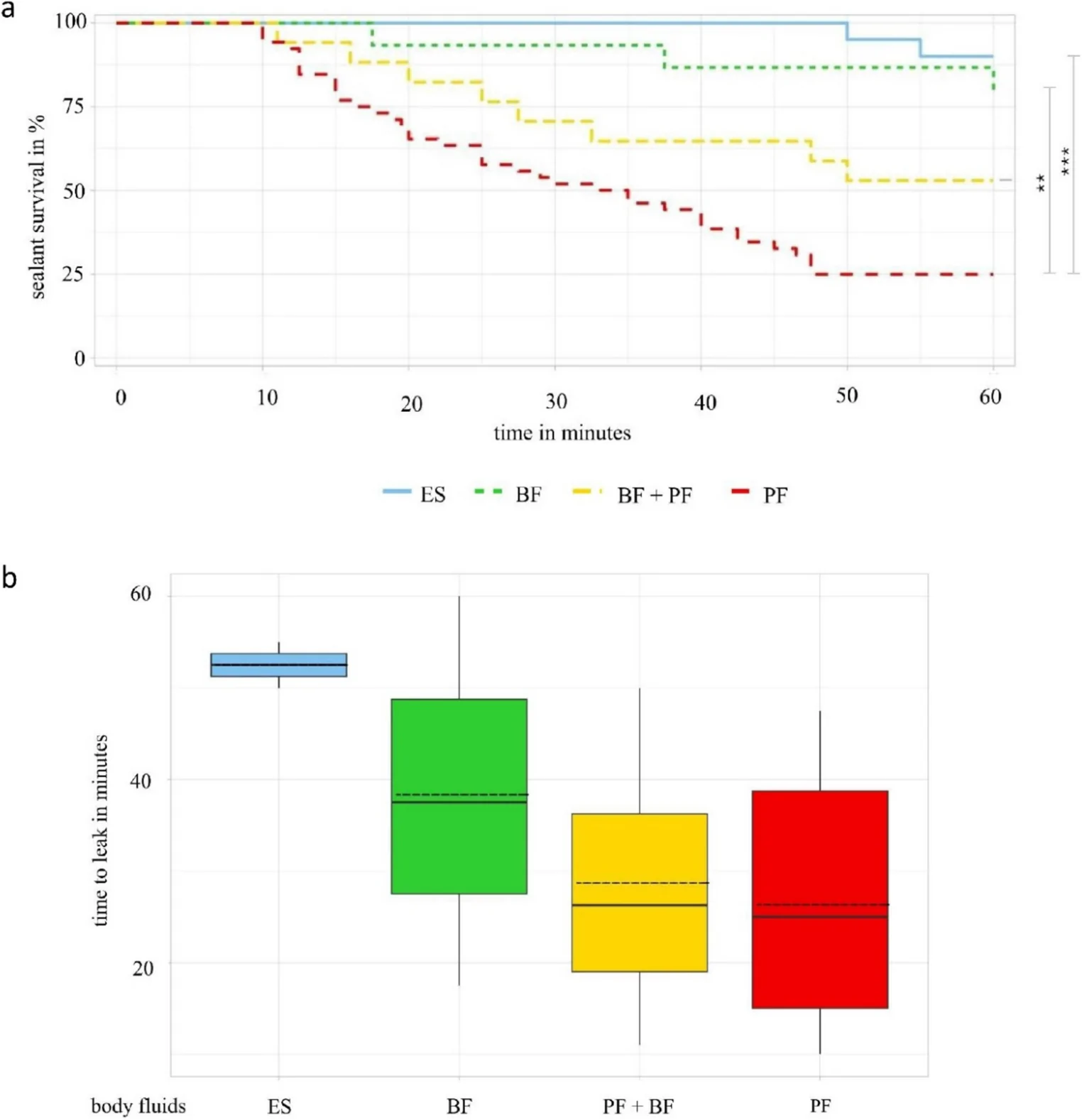
**a**) Kaplan-Meier curves of different body fluids in the PIBS 2. The Kaplan – Meier curves represent the integrity of the fibrin sealant TachoSil^®^ in the PIBS 2 over 60 minutes of incubation with ES = electrolyte solution, BF = bile fluid, PF = pancreatic fluid, PF + BF = combination of pancreatic and bile fluid. Significance levels ** *p* ≤ 0.01, *** = *p* ≤ 0.001. Leakages within the first 5 min were classified as dropouts. Therefore, the Kaplan–Meier curves do not display any events within the initial 5 min interval. **b**) Boxplot of the TTL of a fibrin sealant following exposure to different body fluids in the PIBS 2. The boxplots display the TTL (time to leakage) in minutes of the fibrin sealant TachoSil^®^ being exposed to different body fluids in the PIBS 2 for 60 minutes. ES = electrolyte solution, BF = bile fluid, PF = pancreatic fluid, PF + BF = combination of pancreatic and bile fluid

The highest leakage was measured in PF: evaluation of ten different pancreatic samples in a total of 52 measurements showed a leakage rate of 75%, with an average time to leakage (TTL) of 26.32 min. In contrast, exposure to BF resulted in the lowest number of leakages. BF showed signs of leakage in 3 of the 15 setups (20%), with an average onset after 38.33 min. Combining both fluids (PF + BF) resulted in a leakage in 47.06% (*n* = 8) of measurements, with a TTL after 28.69 min (Fig. 3b).

Figure 3a illustrates the corresponding Kaplan-Meier curves, representing the survival probabilities (stable sealing) over time (in minutes). According to the exclusion criteria, leakage within the first 5 min were excluded from analyses and thereby no events are represented within the first 5 min. A global log-rank test revealed a significant difference of leakages across the tested body fluids (χ² = 33.6, df = 3, *p* < 0.001), with PF contributing most strongly to the observed group differences. For pairwise comparisons, Bonferroni-adjusted p-values were applied (α = 0.0167) (Table 3). Comparisons involving PF consistently demonstrated higher leakage rates and shorter TTL than BF and ES. Specifically, PF showed significantly worse outcomes compared with ES (Fisher’s Exact *p* < 0.001; OR 0.04, 95% CI 0.00–0.20; log-rank *p* < 0.001) and BF (*p* < 0.001; adjusted *p* = 0.001; OR 0.09, 95% CI 0.01–0.39; log-rank *p* < 0.001), with large effect sizes (Cramér’s V = 0.558 and 0.437, respectively). No significant differences were observed between ES and BF (Fisher’s Exact Test, *p* = 0.631, log-rank: 0.392). Analysis of leakage rates using Fisher’s Exact Test confirmed these findings. The combination of PF and BF(PF + BF) also resulted in significantly poorer leakage outcomes compared with ES (unadjusted Fisher’s Exact *p* = 0.023; log-rank *p* = 0.007). After Bonferroni correction, significance was retained for the log-rank analysis (adjusted *p* = 0.028), while the Fisher’s Exact test showed a non-significant trend. The effect size for this comparison was moderate (Cramér’s V = 0.355). Similarly, the comparison between PF and PF + BF showed a statistically significant difference (unadjusted Fisher’s Exact *p* = 0.041), which did not remain after correction (*p* = 0.243). A trend towards a longer TTL in the PF + BF group was observed in the unadjusted log-rank analysis (*p* = 0.039). 

**Table 3:** Comparison of leakage outcomes across body fluids groups. The table summarizes the results of Fisher’s Exact and log-rank tests for all pairwise comparisons of electrolyte solution (ES), pancreatic fluid (PF), bile fluid (BF), and their combination (PF+BF). For each comparison, both the unadjusted p-values and the Bonferroni-corrected p-values are provided to account for multiple testing. Effect sizes are reported as odds ratios (OR) with 95% confidence intervals (CI) and Cramér’s V for Fisher’s Exact test, and median time to leakage (TTL) for log-rank analysis. Dropouts due to a leakage within the first 5 minutes are excluded. The global log-rank show significant differences between groups (χ² = 33.6, df = 3, p < 0.001). For pairwise comparisons, Bonferroni-adjusted p-values were applied (α = 0.0167). Significance levels * = *p* ≤ 0.05, ** = *p* ≤ 0.01, *** = *p* ≤ 0.001

comparison of fluid type	n	Fisher’s Exact p (unadj / adj)	OR (95% CI)	Cramers V	Log-rank p(unadj / adj)
ES vs. BF	ES = 20BF = 15	0.631 / 3.788	2.20 (0.22–30.04)	0.059	0.392 / 1.568
ES vs. PF+BF	ES = 20PF + BF = 17	0.023* / 0.138	0.13 (0.01–0.85)	0.355	0.007** / 0.028*
ES vs. PF	ES = 20PF = 52	< 0.001***	0.04 (0.00–0.20)	0.558	< 0.001***
PF vs. BF	PF = 52BF = 15	< 0.001*** / 0.001**	0.09 (0.01–0.39)	0.437	< 0.001***
BF vs. PF+BF	BF = 15PF + BF	0.148 / 0.885	0.29 (0.04–1.68)	0.218	0.095 / 0.38
PF vs. PF+BF	PF = 52PF + BF = 17	0.041* / 0.243	3.31 (0.92–12.29)	0.222	0.039* / 0.156

Overall, PF demonstrated the highest aggressiveness with respect to sealant failure, whereas BF alone showed the most favourable leakage profile. Contrary to the initial assumption that combining both substances might potentiate sealant degradation, the PF + BF group did not demonstrate a higher leakage rate or shorter time to leakage compared with PF alone.

### Microbiological findings

Microbiological analysis of the intraoperative DHC swabs showed no bacterial growth in approximately half of the samples, while the remaining samples revealed bacterial colonization. The most frequently detected organisms included Klebsiella oxytoca, Serratia liquefaciens, Streptococcus anginosus, Enterobacter cloacae complex, Escherichia coli, and Enterococcus faecalis. Coagulase-negative staphylococci, Enterococcus malodoratus, and Clostridium perfringens were detected less frequently. No 3-MRGN or 4-MRGN organisms were identified. Detailed information about are stated in Supplementary Table 2. All swabs obtained from pancreatic fluid were negative.

## Discussion

Postoperative pancreatic fistulas are among the most feared complications following pancreatic surgery [1, 12]. Due to the rise in postoperative morbidity and mortality, as well as an increased strain on the healthcare system [3, 4, 12], effective preventive and therapeutic strategies remain a critical focus of ongoing research. Preclinical models that reliably reflect the underlying pathophysiological mechanisms are therefore essential. To meet this demand, the experimental model was further refined to facilitate more precise and efficient experimental evaluation in POPF research [17]. The present study was designed to validate an optimized experimental setup (PIBS 2) and to investigate the potential influence of BF on the aggressiveness of PF. TachoSil^®^, a fibrin-based sealant, has shown limited effectiveness in preventing POPF, which has been attributed to the degradation of its fibrin matrix by proteolytic enzymes presented in PF [14]. Given the high enzymatic activity of PF, which is recognized as a risk factor for tissue damage in POPF [1], we hypothesized that BF might enhance the proteolytic activity of PF through enzymatic interactions. However, this assumption could not be confirmed in this present short-term ex-vivo experiment.

### Experimental setup – PIBS 2

The experimental design of this study is based on an innovative ex-vivo physiological incubation bio-simulator (PIBS) model [17]. Modifications of the PIBS 2 represent a technically robust ex-vivo platform that enables standardized short-term exposure of sealing materials to different fluids under controlled temperature, humidity and pressure conditions. The incubation period of 60 min was retained from the established experimental protocol and was considered appropriate for the objectives of the present study, which focused primarily on the initial response of the sealing materials and early fluid–sealant interactions under the defined experimental conditions. However, this timeframe does not allow conclusions to be drawn regarding the long-term stability or durability of the sealants. Longer incubation periods can be implemented with the PIBS depending on the specific research question. In the present study, the established 60-minute period was therefore maintained while the experimental setup was already technically optimized, avoiding simultaneous changes to both the system configuration and the experimental duration. This approach allowed the evaluation of the materials under standardized and controlled conditions while focusing on their initial sealing performance.

The use of porcine pleura as a biological membrane was a deliberate methodological choice balancing reproducibility, ethical considerations and mechanical stability. The histological and biomechanical properties of porcine pleura, particularly its elasticity, are similar to those of human, ensuring a high degree of transferability [18]. During the experimental period porcine pleura showed no sign of autolysis, allowing reproducible assessment of sealant integrity. However, it must be emphasized that this model cannot replicate the biological, mechanical, immunological, or microbiological *in vivo* complexity. Clinically relevant factors such as patient age, comorbidities, tissue frailty, bacterial contamination, and surgeon experience cannot be addressed or excluded in this setup.

To ensure that the observed leakages were due to fluid-sealant interactions and not setup errors, a series of initial experiments were conducted using ES. The 10% leakage rate observed matched expectations based on previous PIBS testing [17], confirming stable defect closure by the sealant in the absence of aggressive fluids. Fisher’s exact test showed no significant differences in leakage rates across the three groups (Fig. 2), validating the stability and reliability of the experimental setup.

While pancreatic swabs appeared sterile, bacterial colonization of the biliary tract may represent an additional confounding factor when assessing the effect of bile on fibrin-based sealant integrity. In our cohort, approximately half of the bile samples were culture-negative, whereas the remaining samples showed colonization with various bacterial species, including Enterococcus spp., Enterobacterales, Streptococcus anginosus, Serratia liquefaciens, and Clostridium perfringens. Differences in the bacterial composition of bile may therefore have contributed to interindividual variability in the observed effects. However, this potential confounder is difficult to address in the clinical setting, as biliary colonization is common in patients undergoing preoperative biliary drainage with a common bile duct (DHC) stent. Consequently, a clinically representative comparison between sterile and non-colonized bile is hardly feasible in this patient population.

Moreover, the primary aim of this study was to investigate the direct chemical interaction between pancreatic and biliary secretions. It was designed to determine whether their combination enhances the fibrinolytic activity against fibrin-based sealants, rather than characterizing the interaction between microorganisms and the sealant. Thus, although a potential influence of biliary bacteria on sealant degradation and fistula formation cannot be excluded, the present data do not allow us to determine whether bacterial colonization affected the observed results. Future studies using standardized, microbiologically defined bile preparations may help to further elucidate this potential interaction.

### Degradation-related effects of various liquids on fibrin-based sealant

This study investigated the effects of BF on the aggressiveness of PF in an ex-vivo setting in a short-term analysis of 60 min. A fibrin-based sealant was used to investigate whether BF exacerbates the deleterious effects of PF on sealant integrity, potentially contributing to the development of fistulas. To confirm the biological activity of the PF, enzymatic activity of amylase, lipase and carboxypeptidase were measure. Enzymatic activity was detected in every sample, confirming that active pancreatic secretions were used for the experimental setup.

We acknowledge that several leakages occurred within the first 5 min after test initiation. Based on predefined criteria [17], these events were classified as technical failures (dropouts) as the contact time between the sealant and fluid was deemed too short for relevant proteolytic effects to occur and were therefore treated as censored observations. To reliably determine the leakage pattern, several measurements were carried out for each fluid sample. This approach allowed us to verify reproducibility and obtain uniform results regarding the type and timing of leakage associated with each fluid sample. After exclusion of the dropouts, the earliest physiological leakage occurred at 26.32 min (PF), 28.69 min (PF + BF), 38,33 min (BF), and 52.50 min (ES) (Table 2; Fig. 3b). Thus, the early < 5-minute events were clearly separated from the main leakage time distribution and did not overlap with the physiological failure times. The positive control showed a mean time to leakage of 20.75 min with a standard deviation of 12.11 min, occurring earlier than any of the sample measurements, which confirms that the system reliably detects leakage. However, the exclusion of early leakages may have an influence on the shape of the Kaplan–Meier curves and biased absolute TTL estimates toward longer durations. Although dropouts were in all experimental groups, this limitation restricts the validity of the time-to-event comparisons.

Exposure to PF resulted in the most pronounced impairment of sealant integrity, with overall high leakage rates and short TTL. A significant negative impact of PF on the integrity of TachoSil^®^, makes its use for securing pancreatic anastomosis and preventing biochemical leakage or POPF appear questionable. These results are consistent with current literature describing PF as an aggressive fluid, rich in digestive enzymes that can degrade tissue and sealing material. These proteolytic enzymes of the PF may be the reason for the degradation and instability of the fibrin matrix of TachoSil^®^ [12, 14]. Clinical studies have also failed to show a reduction in the incidence of POPF using fibrin sealant patches [15, 20]. In addition to the lowest survival rate, the shortest average TTL (26.32 min) was observed in the presence of pure PF. Since the application of a sealant is intended to reduce biochemical leaks and the overflow of pancreatic juice, particularly in the critical phase within the first few days after surgery, this temporal aspect makes their use doubtful as well [12, 21]. However, this study generally reflects only the short-term interactions between the fluid and sealant.

As BF contains enzymes such as tissue-type plasminogen activator, which promote the lysis of fibrin clots [14], an instability of the fibrin sealant towards BF and the combination of PF and BF was assumed. However, the results of these studies showed the opposite: BF showed minimal effects on sealing integrity, indicating that it may be less aggressive and compromising to the sealant than PF under the same conditions. A potentiating effect of BF on PF has been attributed to the alkaline pH of BF, which facilitates the conversion of trypsinogen to trypsin by enteric enterokinase, and lipopolysaccharides presented in contaminated bile, thereby increasing pancreatic enzyme activity [7]. Therefore, the combination of both fluids was hypothesized to result in mutual potentiation, which would be reflected in the highest leakage rate of TachoSil^®^. Our results indicate that, within the 60 min ex-vivo assay, BF did not enhance PF aggressiveness. On the contrary, the combination of PF and BF resulted in lower leakage rates than PF alone (*p* = 0.041). Combining both fluids may result in dilution of the PF and the effective concentration of pancreatic enzymes, which alone may account for reduced degradative effects. Therefore, one could speculate about the dilutive effect of BF and a better clinical outcome when both fluids come in contact *in vivo*. However, the coexistence of both fistulas is associated with an increase in the overall clinical impact, leading to increased morbidity and mortality [7, 8, 16]. This leads to the assumption that clinical outcomes may depend less on the enzymatic interactions here investigated and more on multifactorial influences. Especially BF infections have increasingly become the focus of attention as a risk factor for POPF B and C after PD [4]. Associations between biliary infections, which may be due to preoperative biliary drainage, and POPF after PD have been reported [19]. Since 70% of patients with pancreatic cancer show some degree of biliary obstruction at diagnosis, a perioperative biliary stent is implanted [22]. These conditions are associated with a higher risk of bacterial contamination of the bile duct and may result in postoperative infectious complications after PD [22]. Furthermore, a clinical trial highlighted the link between biliary bacteria, perioperative antibiotics, and complications after PD. Postoperative infections and POPF were associated with cefoxitin-resistant organisms (Enterobacter and Enterococcus) in biliary cultures. The association depended on the type of perioperative antibiotic, occurring only in patients who received cefoxitin and not the broad-spectrum antimicrobial piperacillin-tazobactam [23]. Finally, the reciprocal effects of the intestine and liver can be named. Zhang et al.l described an enterohepatic circulation in which intestinal infections cause modifications of the BF composition, leading to a modified intestinal function [24]. Overall, bacterial contamination of BF and inflammatory processes are risk factors for POPF.

Overall, our results indicate that the combination of PF and BF does not lead to an increased leakage compared with PF alone, suggesting that simple additive effects of the fluids are unlikely to fully explain the high morbidity observed in patients with combined fistulas. However, this study assessed short-term fluid–sealant interactions, and the relatively small and uneven sample sizes limit a generalizability of the reported odds ratios. Instead of enzymatic reinforcement, the clinical impact of a combined fistula may be due to more complex interactions including bacterial contamination and inflammatory responses. Thus, our results align with and support previous observations reported in the literature.

### Limitations and future research

The PIBS 2 model represents an innovative, standardized, and economically feasible experimental setup in compliance with the 3R principle. Modifications allow stable and precise replication of the in-vivo environment, such as temperature, humidity and air pressure. However, this ex-vivo model cannot replicate the full biological complexity of the human body. Critical physiological factors including blood flow, tissue perfusion, immune response, and tissue healing are not represented. Structural differences between porcine pleura and pancreatic anastomotic tissue further limit direct clinical transferability. The absence of blood components may be particularly influential, given the primary design of TachoSil^®^ as a hemostatic agent. Consequently, the findings should be regarded as pre-clinical and hypothesis-generating rather than definitive evidence of clinical efficacy.

Human samples of PF and BF were obtained from different patients, introducing inherent biological and interindividual variability. Furthermore, contamination of the samples by blood or intraoperative irrigation fluid must be recognized.

Limited availability of BF resulted in uneven group sizes and unbalanced sample distribution across experimental condition. In total, 121 tests were performed, of which 17 were characterized as dropouts and excluded from further statistical analysis. No a priori sample size or power calculation was performed, as this study was designed as an exploratory ex-vivo proof-of-concept. An unbalanced sample distribution and dropout rate reduces statistical power and comparability between groups. The resulting odds ratios therefore show unstable point estimates with wide confidence intervals, and the strength of this association is limited. Accordingly, no definitive conclusions regarding a protective or antagonistic effect of BF on PF-induced sealant degradation can be drawn from these data. A low level of significance between PF and PF + BF also suggests that the sample size of the body fluid should be increased uniformly to improve the robustness of the results and provide greater clarity about the interactions between the fluids.

Another aspect is a potential dilution effect when combining PF and BF, which may influence the effective concentration of enzymes and thereby sealant degradation. If bile acted solely as a diluting factor patients with dual fistulas would theoretically have better outcomes, which is not observed clinically. This discrepancy indicates that the high morbidity of combined fistulas is likely driven by other factors. To assess a dilution impact future study should compare PF + ES and PF + BF under equivalent enzyme concentrations.

Finally, the experimental assay was limited to a 60 min observation period, which was adopted from the previously published PIBS model [17] to maintain methodological consistency with the established experimental procedure. The present study focused on the initial response of the sealants and early fluid–sealant interactions under controlled ex-vivo conditions. This observation period allowed the detection and characterization of early sealant responses; however, it does not permit conclusions regarding the persistence or evolution of these effects over the subsequent postoperative period. This limitation is particularly relevant because postoperative pancreatic fistulas (POPF) typically develop within the first three days after surgery [21]. Therefore, the present findings provide short-term mechanistic insights but cannot address longer-term sealant performance or degradation or persistence. Furthermore, this approach does not fully reflect the dynamic clinical development of fistulas, in which a bile fistula may become apparent before the development of a pancreatic fistula. While this experimental design allowed us to directly assess the combined effect of both secretions, it does not account for potential temporal differences in their clinical appearance. Future experiments could therefore investigate sequential exposure, for example by pre-treating the membrane with bile before subsequent exposure to pancreatic juice, to better reflect the dynamic development of fistulas in the clinical setting and provide additional insights into the temporal effects of these secretions on membrane integrity.

Future studies could should therefore extend the observation period and investigate time-dependent interactions to further to characterize the temporal evolution of fluid–sealant interactions and to more closely reproduce the dynamic postoperative pancreatic environment.

Based on our findings, further ex-vivo studies on POPF may focus on three aspects: Improvement of the experimental setup, allowing for more reliable and closer simulation of postoperative pancreatic situations over extended observation periods. Evaluating the effect of specific PF and BF compositions (enzyme activity, bacterial contamination, bicarbonate concentration). Preclinical identification of surgical sealant candidates, which may be able to withstand PF.

## Conclusion

The experimental setup of PIBS 2 was determined to be stable and reliable. These adjustments allowed for an unaffected and standardized experimental setting for testing sealants in a physiological environment.

The study demonstrates that pancreatic fluid substantially compromises the integrity of fibrin sealants under controlled ex-vivo conditions. Contrary to clinical experience, the addition of bile fluid did not increase leakage when combined with pancreatic fluid. These findings underscore the need for future studies incorporating detailed biochemical analyses, microbiological factors, and extended observation periods to more closely replicate in-vivo conditions. Such investigations are essential to bridge the gap between experimental models and clinical reality, ultimately supporting the development of preventive strategies to improve surgical outcomes.

## Supplementary information

Below is the link to the electronic supplementary material.

Supplementary File (DOCX 17.6 KB)

## Data Availability

All data generated or analyzed during this study are included in this article. Further inquiries can be directed to the corresponding author.
