# Supplementary material for: Bile does not increase the aggressiveness of pancreatic fluid towards fibrin-based sealants in a short-term ex-vivo setting
Source: Langenbecks Arch Surg. 2026 Oct 6;411(1):265. doi: 10.1007/s00423-026-04300-2 (PMC13645887; doi:10.1007/s00423-026-04300-2)
Supplement: Supplementary file 1 [file 423_2026_4300_MOESM1_ESM.docx]

**Supplementary Table 1. : Experimental results and enzymatic activity of tested fluids**
The table summarizes the experimental results of the different fluid types and the according mean area under the curve (AUC) and time to leakage (TTL in minutes). The enzymatic activities of amylase, lipase, and carboxypeptidase, as measured photometrically, were presented for the respective pancreatic samples (in mU/ml).

| **fluid type** | **Ø AUC** | **Ø TTL** | **amylase** | **carboxypeptidase** | **lipase** |
| --- | --- | --- | --- | --- | --- |
| PF | 8,742 | 37,5 | 9,124 | 644,174 | 5,573 |
| PF | 41,598 | 28,33 | 3,33 | 81,72 | 6,112 |
| PF | 79,042 | 33,33 | 7,944 | 89,89 | 7,865 |
| PF | 81,766 | 27,5 | 6,441 | 1481,183 | 31,86 |
| PF | 122,396 | 16 | 6,938 | 479,164 | 3,107 |
| PF | 8,194 | 38,75 | 9,55 | 940,343 | 25,568 |
| PF | 150,297 | 20,53 | 2,806 | 32,66 | 41,199 |
| PF | 21,985 | 30 | 108,248 | 241,058 | 7,311 |
| PF | 146,044 | 19,21 | 21,286 | 447,263 | - |
| PF | 75,742 | 30 | 28,553 | 738,673 | 23,096 |
| BF | 0 | 0 |  |  |  |
| BF | 0 | 0 |  |  |  |
| BF | 50,654 | 17,5 |  |  |  |
| BF | 0 | 0 |  |  |  |
| BF | 0,021 | 60 |  |  |  |
| BF | 0,430 | 37,5 |  |  |  |
| PF + BF | 111,517 | 30,33 |  |  |  |
| PF + BF | 52,963 | 21,75 |  |  |  |
| PF + BF | 58,768 | 50 |  |  |  |
| PF + BF | 62,845 | 20 |  |  |  |
| PF + BF | 20,939 | 25 |  |  |  |

**Supplementary Table 2. : Experimental results of the microbial analysis of bile swabs**
The table summarizes the result of the microbial analysis of used bile fluids (low bacterial count (+); moderate bacterial count (++); high bacterial count (+++)).

| **fluid type** | **Culture Test Results** |
| --- | --- |
| BF | coagulase-negative staphylococci (+) |
| BF | Klebsiella oxytoca (++), Serratia liquefaciens (++), Streptococcus anginosus (++), Enterococcus malodoratus (++), Clostridium perfringens (+) |
| BF | No viable microorganisms detected |
| BF | No viable microorganisms detected |
| BF | Enterobacter cloacae complex (++) (no 3-MRGN/4-MRGN), Escherichia coli (++),Enterococcus faecalis (++) |
| BF | No viable microorganisms detected |
